# Effects of Micro-Topography on Soil Nutrients and Plant Diversity of Artificial Shrub Forest in the Mu Us Sandy Land

**DOI:** 10.3390/plants14142163

**Published:** 2025-07-14

**Authors:** Kai Zhao, Long Hai, Fucang Qin, Lei Liu, Guangyu Hong, Zihao Li, Long Li, Yongjie Yue, Xiaoyu Dong, Rong He, Dongming Shi

**Affiliations:** 1 State Key Laboratory of Water Engineering Ecology and Environment in Arid Area, Inner Mongolia Agricultural University, Hohhot 010010, China; zhaokmail@163.com (K.Z.);; 2 College of Desert Control Science and Engineering, Inner Mongolia Agricultural University, Hohhot 010010, China; 3 Inner Mongolia Academy of Forestry Sciences, Hohhot 010011, Chinanmghgy@163.com (G.H.); nmglkylzh@163.com (Z.L.); 4 College of Forestry, Inner Mongolia Agricultural University, Hohhot 010010, China; wolongyue123@163.com (Y.Y.);

**Keywords:** micro-topography, plantated shrublands, soil nutrients, plant diversity, ecological restoration strategies, Mu Us Sandy Land

## Abstract

In ecological restoration of arid/semi-arid sandy lands, micro-topographic variations and artificial shrub arrangement synergistically drive vegetation recovery and soil quality improvement. As a typical fragile ecosystem in northern China, the Mu Us Sandy Land has long suffered wind erosion, desertification, soil infertility, and vegetation degradation, demanding precise vegetation configuration for ecological rehabilitation. This study analyzed soil nutrients, plant diversity, and their correlations under various micro-topographic conditions across different types of artificial shrub plantations in the Mu Us Sandy Land. Employing one-way and two-way ANOVA, we compared the significant differences in soil nutrients and plant diversity indices among different micro-topographic conditions and shrub species. Additionally, redundancy analysis (RDA) was conducted to explore the direct and indirect relationships between micro-topography, shrub species, soil nutrients, and plant diversity. The results show the following: 1. The interdune depressions have the highest plant diversity and optimal soil nutrients, with relatively suitable pH values; the windward slopes and slope tops, due to severe wind erosion, have poor soil nutrients, high pH values, and the lowest plant diversity. Both micro-topography and vegetation can significantly affect soil nutrients and plant diversity (*p* < 0.05), and vegetation has a greater impact on soil nutrients. 2. The correlation between surface soil nutrients and plant diversity is the strongest, and the correlation weakens with increasing soil depth; under different micro-topographic conditions, the influence of soil nutrients on plant diversity varies. 3. In sandy land ecological restoration, a “vegetation type + terrain matching” strategy should be implemented, combining the characteristics of micro-topography and the ecological functions of shrubs for precise configuration, such as planting *Corethrodendron fruticosum* on windward slopes and slope tops to rapidly replenish nutrients, promoting *Salix psammophila* and mixed plantation in interdune depressions and leeward slopes to accumulate organic matter, and prioritizing *Amorpha fruticosa* in areas requiring soil pH adjustment. This study provides a scientific basis and management insights for the ecological restoration and vegetation configuration of the Mu Us Sandy Land.

## 1. Introduction

Soil nutrients, as fundamental resources for plant growth and development, supply plants with essential mineral elements and organic matter and regulate various physiological processes such as photosynthesis and respiration [1,2]. Concurrently, plants, via root exudates, litter accumulation, and biological nitrogen fixation, feed back into the soil to regulate its nutrient content and distribution, forming a dynamic interaction mechanism [3]. Many researchers have explored the relationship between soil nutrients and plant diversity. Shi et al. found that under different land use types, there is a significant correlation between soil nutrients and plant diversity. As soil nutrient content increases, plant diversity initially rises and then stabilizes [4]. Huang et al. discovered that with increasing soil fertility, plant richness and evenness first increase and then decrease. Soil organic matter, total nitrogen, and alkaline hydrolytic nitrogen are significantly positively correlated with plant diversity [5]. The “plant–soil feedback” theory proposed by Wang has not been adequately verified in sandy land ecosystems, particularly regarding the differential responses under various micro-topographical conditions [6]. Some scholars’ research shows that mixed plantations have significantly higher species richness and evenness than monocultures [7]. β-diversity analysis also indicates more complex niche differentiation in mixed plantations [8]. Overall, existing research mainly focuses on single vegetation types or homogenized habitats [9], overlooking the effects of micro-topography on soil nutrient spatial heterogeneity and its link to plant diversity. While some studies have demonstrated the impact of nutrient supply on species richness by controlling soil nutrient heterogeneity, they have mostly focused on grassland or forest ecosystems [10,11], without fully considering the bidirectional feedback mechanisms under extreme conditions in sandy land ecosystems. However, there is less research on the interaction between soil nutrients and plant diversity in sandy land ecosystems, as well as on their roles across different landform scales.

During the migration process of sand dunes under different topographic changes to their micro-topography by altering aeolian sand transport, water redistribution, and light conditions, heterogeneous habitats are formed, thereby influencing soil nutrient distribution and plant community assembly [12,13]. Studies have shown that windward slopes suffer from surface soil nutrient loss due to wind erosion, while leeward slopes and interdune lowlands are more prone to water and organic matter accumulation, providing relatively suitable micro-environments for vegetation growth [14]. Different micro-topographies exhibit distinct characteristics in soil–vegetation relationships [15]. In terms of soil, research in the Tengger Desert has demonstrated significant variations in soil water content across micro-topographies: interdune lowlands have the highest surface soil water content, leeward slopes show the highest water content in middle and deep soil layers, while windward slopes have the lowest water content at all depths; soil water content displays different fluctuation patterns with changes in slope aspect and position [16]. In terms of vegetation, studies on the Loess Plateau have found that vegetation diversity and stability indices are higher on shady slopes than on sunny slopes, and significantly higher at gully bottoms than on slopes, with obvious differences in vegetation species composition and community structure across slope positions and aspects [17]. However, existing studies predominantly focus on relationships between natural vegetation and micro-topography, while the adaptability of artificial shrub forests across micro-topographies and their mechanisms regulating soil–plant interactions remain unclear—particularly lacking systematic comparisons of functional differences between mixed plantations and pure stands along micro-topographic gradients. Further research is needed on the “topography–forest type–diversity–nutrient” synergistic mechanism.

As a critical ecological barrier in China, the Mu Us Sandy Land not only plays a key role as a windbreak, in sand fixation, and in desertification control but also serves as an important region for regional ecological security and biodiversity conservation [18,19]. In recent years, large-scale ecological restoration projects [20,21] in this region have vigorously promoted the establishment of artificial shrub forests to improve soil structure, enhance vegetation coverage, and restore ecological functions [22,23]. However, the specific effects of different shrub types on soil nutrient accumulation and plant diversity improvement vary across micro-topographic conditions, though these differences remain unclear. Therefore, revealing the interaction mechanism between micro-topography and plantation configuration has become a key scientific question for optimizing ecological restoration models in sandy lands. In-depth research on the impacts of different shrub types on soil nutrients and plant diversity across micro-topographies is of great significance for optimizing ecological restoration strategies and enhancing the efficiency of ecological restoration in the Mu Us Sandy Land.

This study selected pure stands of *Salix psammophila*, *Corethrodendron fruticosum*, *Amorpha fruticosa*, mixed plantation of *S. psammophila* + *C. fruticosum* + *A. fruticosa*, and bare sandy land control in typical areas of the Mu Us Sandy Land. Soil nutrient and plant diversity differentiation patterns, systematic comparisons, and correlation analyses between them were conducted along a micro-topographic gradient of windward slope–slope top–leeward slope–interdune lowland. The aim was to reveal the mechanisms by which different artificial shrubs improve soil nutrients and enhance vegetation diversity across micro-topographies, providing theoretical support and practical guidance for further optimizing desert ecological restoration measures. Based on the above background, we propose three research questions: 1. Do significant differences exist in the regulatory effects of various shrub plantations on soil nutrients across micro-topographic conditions? 2. Is there a clear feedback effect between plant diversity and soil nutrients? 3. Is the mixed plantation model superior to single vegetation models in both improving soil nutrients and enhancing vegetation diversity? The research results can provide a theoretical basis for precise configuration of sandy land plantations and micro-topography-adaptive restoration.

## 2. Results

### 2.1. Species Composition of Different Types of Artificial Shrub Plant Communities Under the Influence of Micro-Topography

Table 1 presents the composition of dominant plants in shrub plantations across different micro-topographies. As indicated in the table, there are distinct differences in plant species and their abundances among various micro-topographies. The windward slope harbors eight plant species belonging to four families (Asteraceae, Poaceae, Amaranthaceae, and Apocynaceae) and eight genera, with life forms comprising three categories: herbaceous perennials, annual herbs, and lianas. The slope top supports 10 plant species within four families (Asteraceae, Poaceae, Amaranthaceae, and Apocynaceae) and 10 genera, also featuring the same three life forms (herbaceous perennials, annual herbs, and lianas). The leeward slope contains 11 plant species across four families (Asteraceae, Poaceae, Amaranthaceae, and Apocynaceae) and 11 genera, with annual herbs being the dominant life form (seven species). The interdune lowlands exhibit the highest plant diversity, hosting 20 plant species from six families (Asteraceae, Poaceae, Amaranthaceae, Fabaceae, Apocynaceae, and Euphorbiaceae) and 19 genera, primarily characterized by herbaceous perennials (herbaceous perennials, 12 species) and annual herbs (annual herbs, seven species), with only one liana species. Moreover, on the windward slope, *A. altaicus* and *S. capillata* are the most widely distributed, occurring in *S. psammophila* monoculture plantations, *A. fruticosa* monoculture plantations, and mixed plantation. At the slope top, *A. altaicus* and *S. capillata* are present in all plot types, while *C. chinense*, *S. faberi*, *E. gmelinii*, and *G. dasyphylla* are exclusively found in *S. psammophila* monoculture plantations. On the leeward slope, *S. collina* is distributed in *S. psammophila* plantations, *C. fruticosum* plantations, and mixed plantations; *S. viridis*, *S. glauca*, and *T. aristata* are only observed in *S. psammophila* monoculture plantations, while *I. linariifolia* occurs solely in mixed plantations. In the interdune lowlands, *A. altaicus* and *S. capillata* have extensive distributions, with Asteraceae and Fabaceae species such as *S. brachyotus* and *O. myriophylla* predominantly concentrated in *S. psammophila* monoculture plantations; Melilotus officinalis is only found in *A. fruticosa* monoculture plantations, and *E. crus-galli* occurs exclusively in *A. fruticosa* monoculture plantations and mixed plantations.

**Figure 1:**
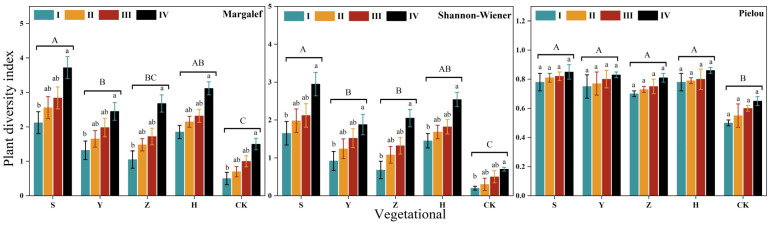
Vegetation species diversity across different vegetation types under micro-topographic variation. Note: S represents *S. psammophila* monoculture; Y represents *C. fruticosum* monoculture; Z represents the monoculture of *A. fruticosa*; H represents S + Y + Z mixed plantation; CK represents bare sand. I indicates windward slope, II indicates slope top, III indicates leeward slope, and IV indicates interdune lowlands. The same letter of a, b and A, B, C indicated no significant difference between groups (*p* > 0.05), and different letters indicated significant difference between groups (*p* < 0.05).

**Figure 2:**
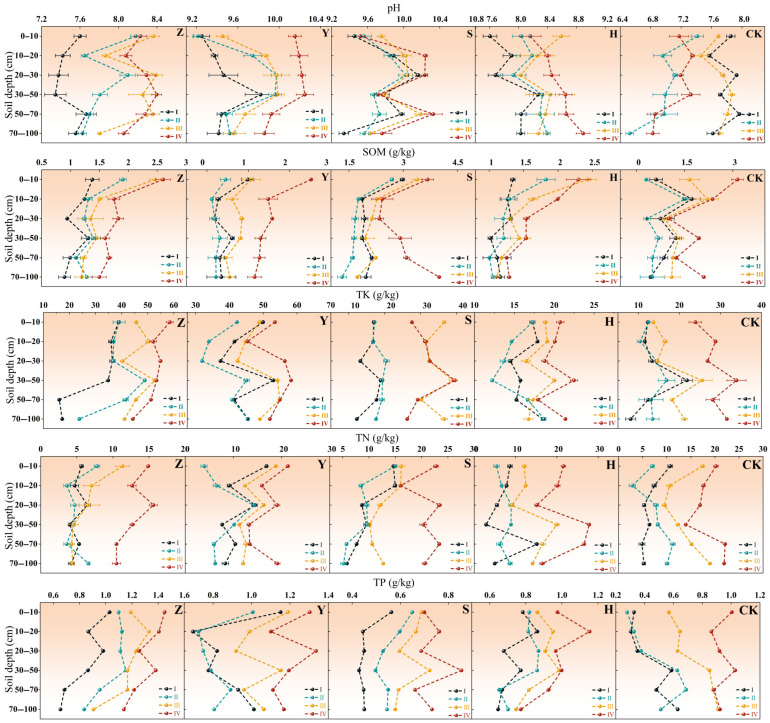
Soil nutrients in micro-topography of the same vegetation type. Note: S denotes *S. psammophila* monoculture, Y denotes *C. fruticosum* monoculture, Z denotes *A. fruticosa* monoculture, H represents S + Y + Z mixed plantation, and CK represents bare sandy land. I indicates windward slope, II indicates slope top, III indicates leeward slope, and IV indicates interdune lowlands.

Figure 1 presents the plant diversity across different vegetation types within various micro-topographies. As shown in Figure 2, plant diversity increases as micro-topography shifts from windward slope to slope top to leeward slope and finally to interdune lowland. The Margalef richness index and Shannon–Wiener diversity index of interdune lowland are significantly higher than those of windward slopes (*p* < 0.05). The Pielou evenness index differs little among micro-topographies but follows the order of interdune lowland > leeward slope > slope top > windward slope. Among the vegetation types, the Margalef and Shannon–Wiener indices of *S. psammophila* monoculture are significantly higher than those of *C. fruticosum* monoculture and *A. fruticosa* monoculture (*p* < 0.05) but show no significant difference from mixed plantation. Moreover, all vegetation types exhibit significantly higher plant diversity than the CK bare sandy land (*p* < 0.05). This indicates that planting any of these plants can significantly enhance plant diversity.

### 2.2. Soil Nutrient Variations Across Micro-Topographies in Different Types of Shrub Plantations

Figure 2 illustrates the variations in soil nutrients across different micro-topographies in various types of shrub plantations. As shown in the figure, distinct differences exist in soil nutrient characteristics under different topographic conditions. The windward slope exhibited overall low soil nutrient contents, particularly for SOM, TN, and TP. Soil pH values ranged from 9.25 to 9.98, indicating strongly alkaline conditions, which may impose certain limitations on plant growth. Specifically, the SOM content on the windward slope ranged from 0.35 to 3.39 g/kg, significantly lower than other micro-topographies, with the lowest SOM observed in *C. fruticosum* monoculture plantations. TN and TP contents were also low, with TN in *C. fruticosum* plantations ranging from 3.63 to 16.88 g/kg. This suggests that windward slope soils are nutrient-poor, and the amelioration effect of vegetation cover on soil nutrients is limited. Soil nutrients at the slope top showed slight improvement compared to the windward slope but remained generally low. Soil pH ranged from 9.56 to 10.24, still strongly alkaline. SOM content varied between 0.18 and 3.68 g/kg, with the lowest SOM in *C. fruticosum* monoculture. TN and TP contents were also low, particularly TN in *C. fruticosum*, which ranged from 3.63 to 19.05 g/kg. This indicates that while slope top soil conditions are superior to windward slopes, they remain insufficient to support full vegetation growth. The leeward slope exhibited better soil nutrient conditions than the windward slopes and slope top, especially in SOM and TN contents. Soil pH ranged from 9.45 to 10.22, still strongly alkaline but less extreme than other micro-topographies. The SOM content ranged from 0.55 to 5.63 g/kg, with the highest value (5.63 g/kg) observed in *S. psammophila* plantations. Higher TN and TP contents indicated that leeward slope soils are more suitable for vegetation growth. Interdune lowlands had the highest soil nutrient levels, particularly in SOM and TN. Soil pH ranged from 7.17 to 10.15, which is relatively moderate, suggesting a more favorable soil environment for plant growth. The SOM content ranged from 1.01 to 4.00 g/kg, with high TN and TP contents, further confirming that interdune lowlands have optimal soil nutrient conditions, where vegetation cover exerts the most significant soil amelioration effect.

### 2.3. Soil Nutrient Variations in Shrub Plantations Across Different Micro-Topographies

**Figure 3:**
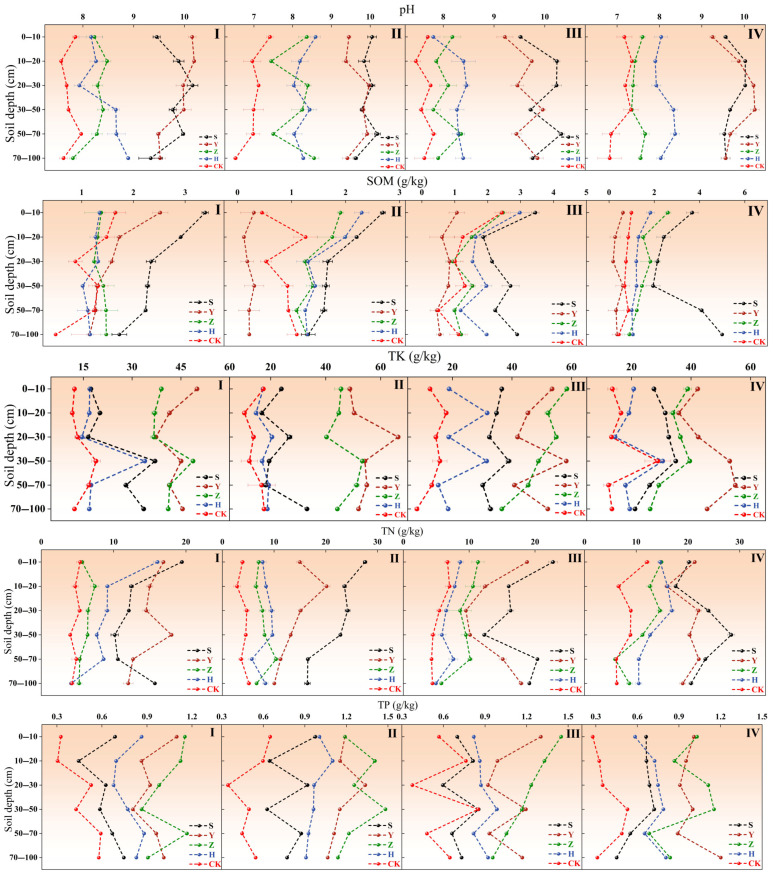
Soil nutrients in micro-topographic vegetation. Note: S denotes *S. psammophila* monoculture, Y denotes *C. fruticosum* monoculture, Z denotes *A. fruticosa* monoculture, H represents S + Y + Z mixed plantation, and CK represents bare sandy land. I indicates windward slope, II indicates slope top, III indicates leeward slope, and IV indicates interdune lowlands.

Figure 3 illustrates soil nutrient variations in different shrub plantation types across micro-topographies. In terms of pH, *S. psammophila* consistently exhibited higher values, particularly in the shallow (0–30 cm) and deep (70–100 cm) soil layers, with a pH of about 9.5. pH showed minimal variation across micro-topographies, remaining predominantly alkaline. *C. fruticosum* also had elevated pH in shallow (0–30 cm) and deep (70–100 cm) layers, slightly lower than *S. psammophila* monocultures, with uniformly high pH across all soil layers in interdune lowlands. In contrast, *A. fruticosa* had significantly lower pH than other vegetation types, especially in the shallow layer (0–30 cm, pH 7.3–8.5). While the pH in the deep layer (50–100 cm) of interdune lowlands increased slightly for *A. fruticosa*, it remained lower than other vegetation types. The mixed plantation had pH values intermediate between *A. fruticosa* and *C. fruticosum*, generally slightly lower than *S. psammophila* monocultures. Bare sandy land (CK) had the lowest soil pH. Overall, *S. psammophila* and *C. fruticosum* monocultures may induce soil alkalization, whereas *A. fruticosa* monocultures and mixed plantations demonstrated stronger regulation of soil pH. For SOM, *S. psammophila* plantations generally had higher SOM content, particularly in the shallow (0–10 cm, 3.68 g/kg) and deep (70–100 cm, 4.00 g/kg) layers of interdune lowlands. The SOM content was relatively lower on windward slopes and slope top but still higher than other vegetation types overall. *C. fruticosum* had a significantly lower SOM than other types, especially in shallow (0–10 cm) and middle (10–30 cm) layers (about 1.00 g/kg). SOM in *A. fruticosa* and mixed plantation fell between *S. psammophila* and *C. fruticosum*, generally slightly lower than *S. psammophila*. In summary, *S. psammophila* and mixed plantations exhibited the strongest SOM accumulation, particularly in interdune lowlands and leeward slopes. *C. fruticosum* monocultures and bare sandy land had a low SOM, necessitating vegetation configuration and soil amendment to enhance soil fertility. Regarding TK, *C. fruticosum* soil had a significantly higher TK than other vegetation types, especially in shallow (0–30 cm) layers and interdune lowlands (TK of about 50 g/kg), with elevated TK also observed on windward slopes and slope top, likely linked to root activity and litter decomposition of *C. fruticosum*. *S. psammophila* had a higher TK in interdune lowlands and leeward slopes, particularly in shallow layers (0–30 cm, TK approaching or exceeding 30 g/kg). *A. fruticosa* soil TK fell between *S. psammophila* and *C. fruticosum*, generally slightly lower than *C. fruticosum*. Bare sandy land (CK) had the lowest TK, especially in shallow layers (0–30 cm, about 15 g/kg). Thus, *C. fruticosum* monocultures demonstrated the strongest TK accumulation, particularly in interdune lowlands and leeward slopes, followed by *A. fruticosa* and *S. psammophila*, with bare sandy land having the lowest TK and mixed plantation only slightly higher than CK. For TN, *C. fruticosum* and *S. psammophila* soils had a significantly higher TN than other types. *C. fruticosum* had a TN of about 20 g/kg in shallow layers (0–30 cm) and interdune lowlands, with relatively lower but still elevated TN on windward slopes and slope top. *A. fruticosa* and mixed plantations had a lower TN than *S. psammophila* and *C. fruticosum* across all micro-topographies but higher than CK. In conclusion, *C. fruticosum* and *S. psammophila* exhibited the strongest TN accumulation, followed by *A. fruticosa*, with bare sandy land having the lowest TN and mixed plantation slightly higher than CK. For total phosphorus (TP), *C. fruticosum* and *A. fruticosa* plantations demonstrated the strongest TP accumulation across all micro-topographies, followed by *S. psammophila* monocultures and mixed plantations, with bare sandy land having the lowest TP content.

A two-way ANOVA was employed to analyze the effects of micro-topography and vegetation type on soil nutrients (pH, SOM, TN, TP, and TK), followed by post hoc tests (*p* < 0.05). Table 2 demonstrates that, with the exception of micro-topography exhibiting a significant correlation with SOM (*p* < 0.05) and TP showing no significant interaction effect between micro-topography and vegetation type, all other soil nutrient factors exhibited highly significant correlations (*p* < 0.01) with both vegetation type and micro-topography. This indicates that both micro-topography and vegetation type independently influence the distribution of soil nutrients. Furthermore, the interaction between micro-topography and vegetation type exerted a highly significant effect on the distribution of all soil nutrients except TP, where it was non-significant. These findings corroborate the patterns observed in Figure 2 and Figure 3. Comparing the F-values among the three sources of variation revealed significant correlations in all cases; however, the F-values for vegetation type were consistently the highest, suggesting that vegetation type exerts a stronger influence on soil nutrients.

Post hoc test results indicate the following: For pH, significant differences existed across micro-topographic positions. Windward and leeward slopes exhibited relatively higher pH values, while the interdune lowland showed lower values. Vegetation types also significantly influenced pH: *S. psammophila* monoculture and *C. fruticosum* monoculture had higher pH, CK had the lowest, and *A. fruticose* monoculture and mixed plantation showed intermediate values. Regarding SOM, windward and leeward slopes contained higher SOM levels than the slope top; *S. psammophila* monoculture had the highest SOM content, while *C. fruticosum* monoculture had the lowest. For TN, the interdune lowland had the highest TN content, the windward slope the lowest, and the slope top was intermediate. Among vegetation types, *S. psammophila* monoculture had the highest TN content, and CK had the lowest. For TP and TK, micro-topographic factors exhibited slightly different patterns, but overall, the interdune lowland and leeward slope contained higher levels than the windward slope and slope top. Among vegetation types, *C. fruticosum* monoculture had the highest levels, while CK had the lowest. In summary, the results of the two-way ANOVA and subsequent post hoc tests demonstrate a high degree of consistency and correlation with the findings presented in Figure 2 and Figure 3.

### 2.4. Correlation Analysis Between Plant Diversity and Soil Nutrients

**Figure 4:**
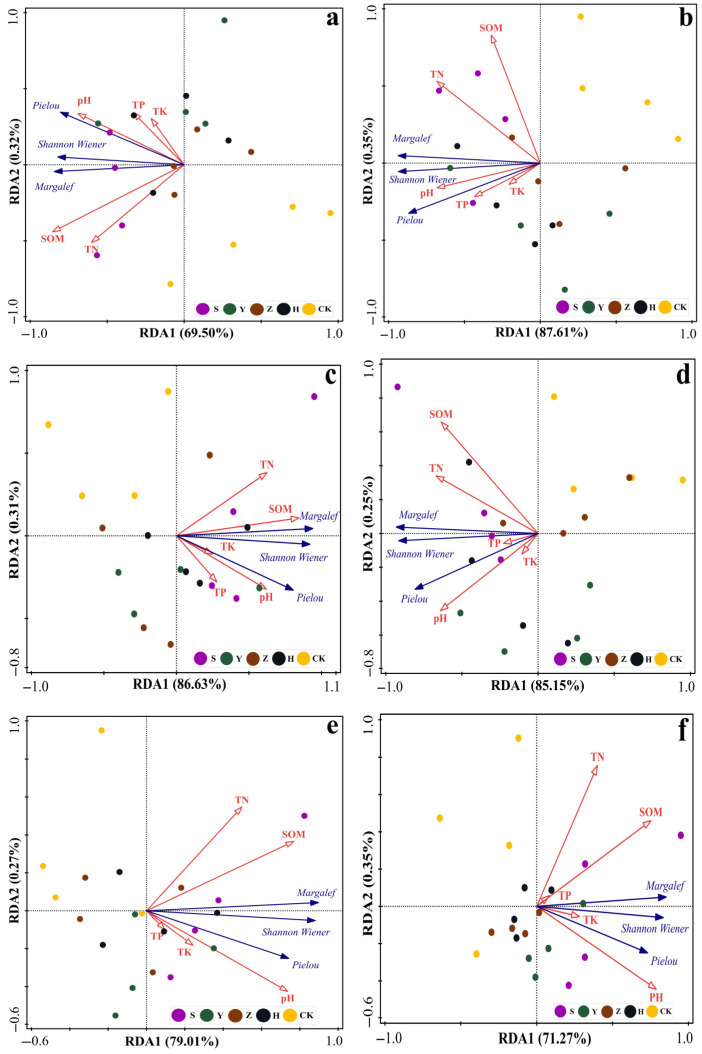
Relationships between soil nutrients and plant diversity across different soil layers. Note: (**a**–**f**) represent soil layers of 0–10 cm, 10–20 cm, 20–30 cm, 30–50 cm, 50–70 cm, and 70–100 cm, respectively. S denotes *S. psammophila* monoculture, Y denotes *C. fruticosum* monoculture, Z denotes *A. fruticosa* monoculture, H represents S + Y + Z mixed plantation, and CK represents bare sandy land.

We conducted redundancy analysis (RDA) on five soil nutrient indices (pH, TP, TK, TN, and SOM) and three diversity indices (Margalef species richness index, Shannon–Wiener diversity index, and Pielou evenness index) across six soil layers (0–10 cm, 10–20 cm, 20–30 cm, 30–50 cm, 50–70 cm, and 70–100 cm) (Figure 4). As shown in the figure, the first and second axes of RDA for different soil layers explained 69.82%, 87.96%, 86.94%, 85.40%, 79.28%, and 71.62% of species diversity, respectively, indicating that RDA1 and RDA2 effectively reflect the influence of soil nutrient factors on species diversity, with the first axis playing a key role. Blue arrows in the figure represent diversity indices, red hollow arrows denote environmental factors, and dots indicate different shrub plots.

RDA results showed that soil nutrients in different soil layers significantly influenced species diversity. Specifically, in the surface soil (0–10 cm), soil nutrient factors such as SOM, TN, TP, TK, and pH exhibited the most pronounced effects on plant diversity. These factors had longer arrows with smaller angles than the plant diversity index arrows, indicating strong positive correlations—higher soil nutrient contents were associated with higher plant diversity and more abundant/diverse plant community structures. As soil depth increased to the middle layers (10–50 cm), the arrows for pH, TP, and TK gradually shortened, reflecting weakened correlations. In deep soil layers (50–100 cm), the TP and TK arrows further shortened, indicating a further decline in the correlation between soil nutrients and plant diversity. Overall, while the correlation between soil nutrients and plant diversity remained positive with increasing soil depth, its strength diminished with soil layer changes. In summary, the soil nutrient indices influencing species diversity were consistent across soil depths, and the impact of soil nutrient factors on plant diversity showed little variation among layers. In terms of plot distribution, surface soil (0–10 cm) plots were relatively concentrated along the RDA1 and RDA2 axes, with most plots positioned in the positive direction of RDA1. This suggests that plant diversity in surface soil was uniformly driven by soil nutrient factors, with strong homogeneity in the influence of soil nutrients on plant communities. Middle soil (10–50 cm) plots became more dispersed, with expanded projection ranges on the RDA1 and RDA2 axes, indicating that plant diversity was increasingly influenced by multiple factors as soil depth increased, with growing differences among plots. Deep soil (50–100 cm) plots were further dispersed, with even broader projections on the axes, reflecting complex multi-factor influences on plant diversity in deep layers, significant differences among samples, and lower explanatory power of soil nutrient factors.

In Figure 4, we observed minimal differences in the impact of different soil layers on vegetation diversity. Therefore, we treated the plots as a whole to analyze the relationships between surface soil nutrients and plant diversity across micro-topographies (Figure 4). As shown, the first and second axes of RDA for different micro-topographies explained 99.97%, 99.99%, 99.98%, and 100% of species diversity, respectively, all effectively indicating the influence of soil nutrient factors on species diversity, with the first axis playing a dominant role.

**Figure 5:**
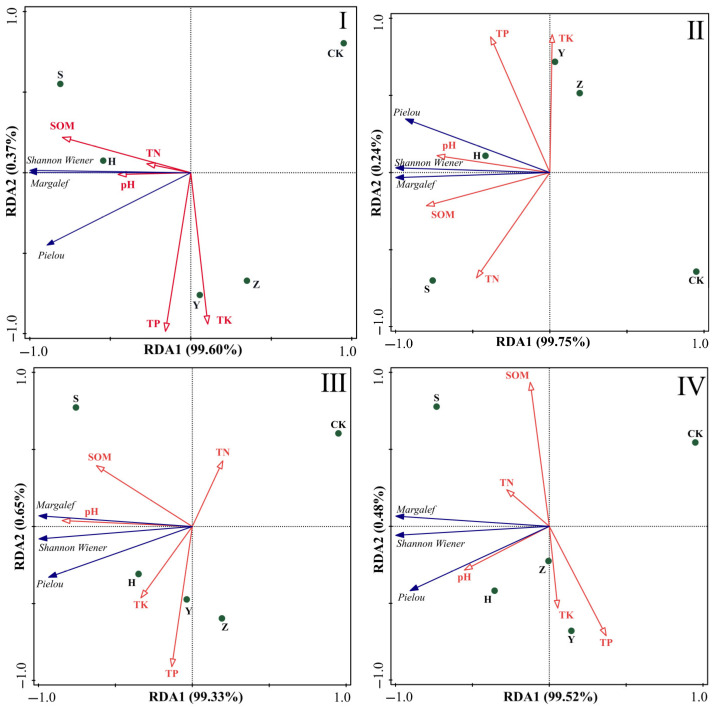
Relationships between micro-topographic soil nutrients and plant diversity. Note: I indicates windward slope, II indicates slope top, III indicates leeward slope, and IV indicates interdune lowlands. S denotes *S. psammophila* monoculture, Y denotes *C. fruticosum* monoculture, Z denotes *A. fruticosa* monoculture, H represents S + Y + Z mixed plantation, and CK represents bare sandy land.

Figure 5 shows that plant diversity across micro-topographies was closely related to soil nutrients, though correlation patterns varied. Plant diversity on windward slopes had significant positive correlations with SOM, TN, and pH, but weak or negative correlations with TK. At the slope top, plant diversity was positively correlated with pH, SOM, TN, and TP, with weak correlation with TK; TN showed negative correlations with TP and TK. On leeward slopes, plant diversity had positive correlations with SOM, TK, TP, and pH, but a negative correlation with TN; TN was highly negatively correlated with TP and TK. In interdune lowlands, plant diversity was positively correlated with TN and pH, showed weak correlations with SOM and TK, and had a negative correlation with TP; total TN and SOM had negative correlations (arrows pointing in opposite directions) with TP and TK.

## 3. Discussion

### 3.1. Impacts of Micro-Topography on Soil Nutrients and Plant Diversity

In this study, plant species richness and soil nutrient contents in interdune lowlands were significantly higher than those in other micro-topographies, while windward slopes and the slope top exhibited lower plant diversity and soil nutrient levels, with leeward slopes being intermediate between them. Li et al. [24] found that during landform development, soil moisture and nutrients were significantly concentrated in interdune depressions due to soil resource redistribution. The soil moisture and nutrient levels at the base of the dune were significantly higher than those on the dune slope and top, which was consistent with our research. There are also research findings indicating that micro-topography shapes differentiated habitat gradients by altering hydrothermal conditions, aeolian activity intensity, and sediment distribution, thereby significantly influencing soil nutrients and plant diversity [25,26]. Windward slopes and the slope top generally had low soil nutrients and high pH, primarily due to long-term aeolian erosion. Steep slopes and wind action caused organic matter and soluble nutrients to be easily lost via erosion and runoff, coupled with poor moisture conditions and low vegetation cover, which limited the return of plant residues to the soil and further constrained nutrient accumulation. The strongly alkaline soil environment also hindered root uptake and utilization of nutrients [27]. In contrast, leeward slopes and interdune lowlands, as catchment areas for water and sediments, benefited from lower slopes and larger catchment areas that facilitated rainfall-runoff deposition, thereby improving soil nutrient conditions; these areas exhibited higher SOM, TN, and TP contents.

In terms of vegetation composition, interdune lowlands harbored the richest plant species with life forms dominated by perennial and annual herbs, indicating that under stable moisture and optimal nutrient conditions, plants tend to adopt conservative, sustained resource-use growth strategies. The high proportion of annual herbs on leeward slopes likely reflects an ecological strategy of rapid reproduction to exploit short-lived resource windows due to limited local resource availability [28]. Micro-topography not only provides diverse habitat conditions but also promotes differentiation in physiological and ecological traits among plant populations [29]. In *S. psammophila* monoculture, plant diversity and soil nutrient distributions on both windward slopes and interdune lowlands showed strong gradient patterns linked to topographic conditions, suggesting that although artificial vegetation configuration directly impacts local ecosystems, micro-topography plays a fundamental role in enhancing or constraining vegetation growth by regulating water, sedimentation, and soil chemistry. Han et al. [30,31] studied the relationship between fixed-dune micro-topography and above-ground vegetation and revealed that with differences in micro-topography, plant species, quantity, coverage, frequency, and above-ground biomass almost invariably follow the sequence of lower slope > lower middle slope > middle slope > upper middle slope > upper slope, which is in agreement with our research. From an applied perspective, incorporating micro-topographic factors in vegetation restoration and ecological engineering can provide a scientific basis for optimizing soil resource distribution and enhancing vegetation diversity. In summary, micro-topography regulates soil nutrient distribution and vegetation diversity patterns by influencing precipitation runoff, sedimentation processes, and soil physicochemical properties. The cumulative effects of water and nutrients in interdune lowlands not only directly improve soil environments but also further enhance overall ecosystem stability through vegetation cover feedback.

### 3.2. Impacts of Stand Types on Soil Nutrients and Plant Diversity

This study revealed significant differences in pH, SOM, TK, TN, and TP contents among different monocultures and mixed plantations (*p* < 0.05), thereby influencing plant diversity and species composition. Different stand types significantly regulate soil nutrient dynamics and plant community assembly across micro-topographies through processes such as litter input, root activity, and interspecific interactions [32,33]. *S. psammophila* monocultures, due to their large canopy and deep root systems, not only accumulate abundant litter to stabilize organic matter formation but may also cause gradual release of alkaline ions from litter rich in lignin and cellulose, leading to persistently high soil pH [34]. Meanwhile, *S. psammophila* efficiently accumulates total nitrogen and potassium by absorbing deep-layer nutrients through its roots and transporting them upward [35]. In contrast, *C. fruticosum* monocultures, with their sparse shrub structure and shallow root systems, rapidly release mineral nutrients via quick litter decomposition, resulting in high TK and TN contents in shallow soils—though this fast-growing trait also entails rapid nutrient depletion and potential alkalization risks [36]. *A. fruticosa* monoculture, by secreting organic acids from roots and releasing protons during nitrogen fixation, significantly reduce rhizosphere and shallow soil pH, creating a more suitable environment for Fabaceae and other acid–base-sensitive plants while promoting phosphorus mobilization and uptake. Mixed plantations leverage species complementarity: *S. psammophila*’s shading, *C. fruticosum*’s rapid nutrient release, and *A. fruticosa*’s pH regulation create a neutral buffering effect, exhibiting intermediate characteristics in soil nutrient accumulation and plant diversity between monocultures.

Moreover, micro-topography further modulates the effects of shrub types on soil physicochemical properties and vegetation communities [37]. In wind-eroded slope tops and windward slopes, the deep-rooted, wind-resistant traits of *C. fruticosum* and *S. psammophila* stabilize soils and rapidly replenish TK and TN, facilitating pioneer species’ establishment. In sediment-rich leeward slopes and interdune lowlands, *S. psammophila* and mixed systems promote SOM and nutrient (TN and TP) accumulation via abundant litter and shading, while *A. fruticosa*’s acidification regulation locally enhances soil nutrient availability. Bare sandy areas sustain only a few stress-tolerant species, highlighting the critical role of shrub vegetation in enhancing regional plant diversity and ecosystem stability. Thus, *S. psammophila* is suitable for rapid SOM accumulation and soil structure improvement but requires attention to potential alkalization; *C. fruticosum* is ideal for quick TK/TN replenishment in nutrient-leaching areas; *A. fruticosa* and mixed plantation excel in pH regulation and moderate nutrient maintenance. Collectively, shrub types exert profound impacts on soil nutrient distribution and microhabitat formation through their unique physiological traits and ecological functions [38].

### 3.3. Correlations Between Soil Nutrients and Vegetation Types

In this study, RDA analysis found that nutrient characteristics in the surface soil (0–10 cm), such as SOM, TN, TP, TK, and pH, exhibited significant positive correlations with plant diversity. This phenomenon reflects the direct input of organic carbon and nutrients to surface soil through vegetation litterfall, root exudates, and fine root activity, establishing it as a critical regulatory layer for plant community distribution and composition [39]. As soil depth increased, the vector lengths of nutrient factors gradually shortened, and their explanatory power for plant diversity decreased, indicating that deep soils are dominated by physical–chemical processes like geological sedimentation and water leaching. The direct influence of plant roots weakens in deeper layers, and slow nutrient migration rates reduce nutrient availability [40]. This vertical differentiation highlights the surface soil as a key regulatory layer for vegetation community assembly, while middle–deep soils primarily function as long-term nutrient reservoirs [41]. Meanwhile, micro-topographic variations further intensified the heterogeneous associations between soil nutrients and plant diversity [42]. On windward slopes and slope tops with severe aeolian erosion, plant diversity was primarily driven by SOM, TN, and pH, with weak or negative effects from TK—this relates to potassium loss due to erosion and alkaline ion enrichment. Shrubs like *S. psammophila* and *C. fruticosum* serve as core nutrient sources in these constrained areas through litter-derived organic matter and nitrogen accumulation. The high-pH environment selects for alkali-tolerant species (e.g., *C. chinense* and *E. gmelinii*), forming species coexistence patterns under specific stress conditions. In sedimentary leeward slopes and interdune lowlands, nutrient effects diverged: SOM, TK, TP, and pH synergistically promoted diversity on leeward slopes, while TN showed inhibitory effects, possibly due to short-term nutrient imbalances from rapid nitrogen consumption by annual herbs. In interdune lowlands, TN and pH were the primary drivers, whereas the negative TP correlation suggested phosphorus excess might induce competitive exclusion of dominant species, reflecting the critical role of N–P balance in shaping community structure under moist microhabitats. These micro-topographic regulatory mechanisms fundamentally arise from the interplay of water–sediment processes, vegetation feedback, and nutrient cycling.

Collectively, the correlations between soil nutrients and plant diversity exhibit a vertical gradient of “surface-dominated, deep-layer decline” and a horizontal micro-topographic pattern of “nutrient limitation in stress environments vs. nutrient balance in sedimentary zones.” Yu’s research found that in semiarid catchments, soil factors from different soil layers have similar effects on plant diversity and above-ground biomass of communities, and that surface soil factors have the most significant impact, which is in line with our research results [43]. As the most dynamically active nutrient zone, surface soil directly and significantly drives plant communities, while the influence of nutrients in middle–deep soils gradually diminishes, with other environmental factors like soil structure and microbial communities potentially playing supplementary regulatory roles. Additionally, water availability, erosion–sedimentation, and nutrient loss processes under different micro-topographies further lead to divergent effects of the same nutrient factors on community composition across regions.

## 4. Materials and Methods

### 4.1. Location and Materials

**Figure 6:**
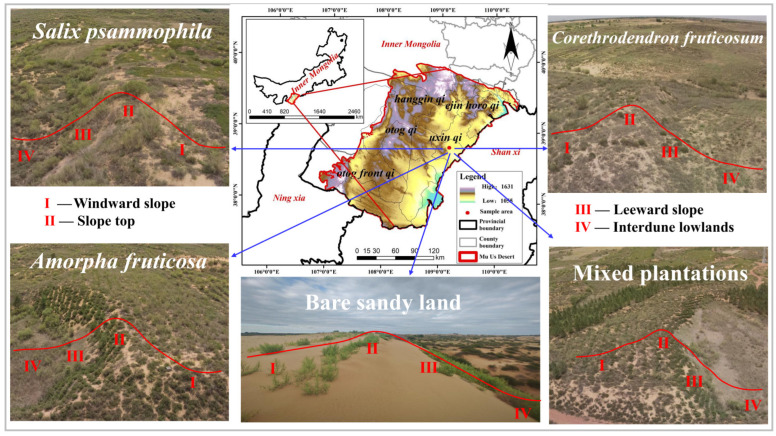
Overview of the study area.

The study area is situated within the multi-year exclosure zone of the Wulantaolegai Sand Control Station in the Mu Us Sandy Land, Wushen Banner, Ordos City, Inner Mongolia (38°61′ N, 108°82′ E, Figure 6). The climate falls under the arid and semiarid continental monsoon regime, with a mean annual temperature of 6.0–8.0 °C, an average annual precipitation of approximately 360 mm (over 70% of which is concentrated in August–September), and annual evaporation ranging from 2200 to 2800 mm. Soil types include aeolian soils in fixed, semi-fixed, and mobile sand dunes, as well as meadow soils, marsh soils, and saline–alkali soils distributed in low-lying areas of floodplains and interdune depressions. The soil texture is dominated by sand particles, characterized by infertility, loose structure, and poor water-retention capacity. Vegetation is primarily composed of azonal vegetation types such as psammophytic and meadow communities, with perennial herbs significantly outnumbering annual and biennial herbs. Key constructive and dominant species in the ecosystem include perennial herbs and shrubs such as *Artemisia ordosica*, *Aster altaicus*, *Medicago ruthenica*, and *Stipa caucasica*, while tree species include *Salix matsudana* and *Pinus sylvestris* var. *mongolica*. Mobile sand dunes are predominantly populated by annual herbs like *Agriophyllum squarrosum* and *Leymus secalinus*.

### 4.2. Methods

#### 4.2.1. Sample Plot Setting and Investigation

In the study area, four complete topographic relief units (windward slope, slope top, leeward slope, and interdune lowland) were selected, with artificial shrub plantations established in four modes: *S. psammophila* pure stand, *C. fruticosum* pure stand, *A. fruticosa* pure stand, and *S. psammophila* + *C. fruticosum* + *A. fruticosa* mixed plantation. Additionally, a bare sand dune in a complete topographic unit was selected as the control (CK). For each micro-topography, two 10 m × 10 m plots were established, with three 1 m × 1 m quadrats set along the diagonal of each plot. In total, 32 plots and 96 quadrats were surveyed from July to August 2024. Within each 1 m × 1 m quadrat, species name (for plant identification, we combine three methods to enhance accuracy: (1) consulting floras based on plant characteristics, (2) using specialized software to analyze images, and (3) expert identification), clump diameter, number of clumps, and plant height of each plant were recorded in detail. General characteristics of the plots are shown in Table 3.

#### 4.2.2. Soil Sample Collection and Determination

The soil sampling depth was 100 cm, divided into six layers: 0–10 cm, 10–20 cm, 20–30 cm, 30–50 cm, 50–70 cm, and 70–100 cm. Three replicates were collected for each layer, and fresh soil samples from the same layer were mixed, stored in sealed plastic bags, and transported to the laboratory for determination of pH, soil organic matter (SOM), total nitrogen (TN), total phosphorus (TP), and total potassium (TK). Soil samples were air-dried in a cool, ventilated area, with roots, gravel, and other impurities removed. Depending on the measured indices, air-dried soil samples were passed through 2 mm, 1 mm, and 100-mesh soil sieves. Specific measurement methods were as follows [44]:

(1) pH

Soil samples were mixed with distilled water at a 1:2.5 ratio, extracted for 30 min, and the suspension pH was measured using a pH meter (Mettler Toledo, Shanghai, China).

(2) Soil organic matter (SOM)

The potassium dichromate oxidation–external heating method was used. A 0.5–1.0 g air-dried soil sample was weighed and mixed with 10 mL of potassium dichromate solution and 20 mL of 3 mol/L sulfuric acid. The mixture was heated on an electric stove to boiling and maintained at a gentle boil for 5–10 min. After cooling, 3–4 drops of o-phenanthroline indicator were added, and the solution was titrated with 0.05 mol/L ferrous sulfate until the color changed from blue to green. Blank tests were conducted simultaneously, and the SOM content was calculated based on titration volume and conversion factors.

(3) Total nitrogen (TN)

The Kjeldahl method was employed. A 0.5–1.0 g air-dried soil sample was weighed and mixed with 10 mL of concentrated sulfuric acid and 10 g of a potassium sulfate–copper sulfate mixed catalyst. The mixture was heated on an electric stove until the solution clarified, then it was cooled and transferred to a Kjeldahl distillation unit. A 40% sodium hydroxide solution was added to render the solution alkaline, and ammonia gas was distilled and absorbed by 2% boric acid. After adding methyl red–bromocresol green indicator, the solution was titrated with 0.05 mol/L hydrochloric acid standard solution until the color changed from blue-green to gray-red. TN content was calculated based on hydrochloric acid consumption.

(4) Total phosphorus (TP)

The acid digestion–molybdenum antimony resistance colorimetry method was used. A 0.5–1.0 g air-dried soil sample was weighed and mixed with 10 mL of 1 mol/L hydrochloric acid, heated on a hotplate to boiling, and maintained at a gentle boil for 5 min. After cooling, the solution was filtered into a 50 mL volumetric flask and made up to volume. Next, 5 mL of the filtrate was mixed with 5 mL of molybdenum antimony resistance chromogenic agent, allowed to stand for 10 min for complete color development, and absorbance was measured at 660 nm using a spectrophotometer. TP content was calculated using a standard curve.

(5) Total potassium (TK)

Flame photometry was applied. A 0.5–1.0 g air-dried soil sample was weighed and mixed with 10 mL of 1 mol/L hydrochloric acid, heated on a hotplate to boiling and maintained at a gentle boil for 5 min. After cooling, the solution was filtered into a 50 mL volumetric flask and made up to volume. An appropriate amount of filtrate was diluted to a suitable concentration, and potassium emission intensity was measured using a calibrated flame photometer. TK content was calculated using a standard curve.

#### 4.2.3. Plant Diversity Index Calculation

The calculation formulas for plant diversity indices (Margalef species richness index, Shannon–Wiener diversity index, and Pielou evenness index) are as follows [45,46]:

(1) Margalef species richness index (*R*)

$$R=\left(S-1\right){log}_{2}N$$

where *S* refers to the total number of herbaceous species in the quadrat, and *N* refers to the total number of herbaceous individuals in the quadrat.

(2) Shannon–Wiener diversity index (*H*)

$$H=-\sum_{i-1}^{n}P_{i}\;lnP_{i}$$

where *P_i_* is the abundance ratio of species *i*.

(3) Pielou evenness index (*J*)

J=H/lnS

where *H* refers to the Shannon–Wiener diversity index, and *S* refers to the total number of herbaceous species in the quadrat.

#### 4.2.4. Data Analysis

The study employed two-way ANOVA and post hoc tests in SPSS 22 and Stata 18 to analyze the effects of micro-topography and vegetation types on soil nutrients. Additionally, one-way ANOVA was used to examine variance and test the significance of differences in plant diversity across different micro-topographies and vegetation types. Data organization was performed using Excel (2021), plotting with Origin (2022), and redundancy analysis (RDA) was conducted using Canoco5 software [47,48].

## 5. Conclusions

**Figure 7:**
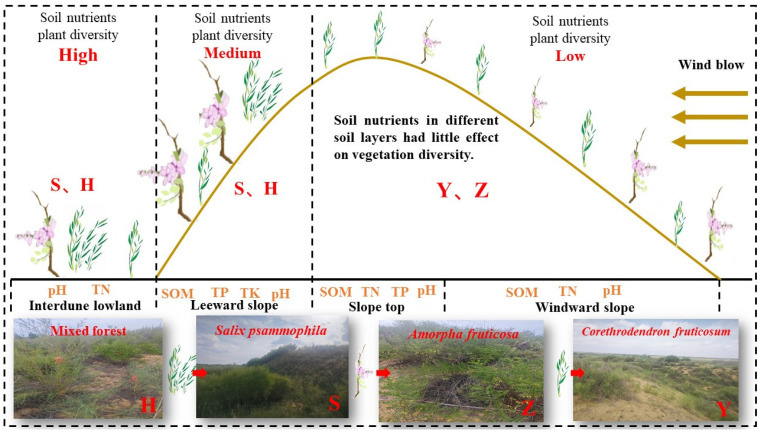
Soil nutrients and plant diversity characteristics of micro-topography in Mu Us Sandy Land, and the suitability of different vegetation types in micro-topography.

In this study, the soil nutrients and plant diversity of micro-topography and shrub types in Mu Us Sandy Land were systematically analyzed (Figure 7). The main conclusions are as follows: 1. Interdune lowlands not only had the highest species richness but also the highest contents of soil SOM, TN, and TP, while windward slopes and slope tops were affected by aeolian erosion and runoff, resulting in poor soil nutrient conditions and low plant diversity, with leeward slopes being intermediate. Vegetation and micro-topography have a significant impact on soil nutrients and plant diversity. 2. *S. psammophila* monocultures are preferentially used in interdune lowlands and leeward slopes, but they need to be intercropped with acid-regulating species such as *A. fruticosa* to alleviate the risk of alkalization; *C. fruticosum* monocultures are suitable for eroded areas such as windward slopes and slope tops; *A. fruticosa* monocultures are used in areas with excessively high pH; mixed plantations are promoted in areas with better hydrothermal conditions. 3. The relationship between soil nutrients and plant diversity showed vertical differentiation characteristics: surface soil nutrients were the key driving factors for plant diversity, with weakened influence as the soil layer deepened; under different micro-topographies, plant diversity had different responses to nutrients. This study reveals the impact of micro-topography on soil nutrients and plant diversity in artificial shrublands.

## Figures and Tables

**Table 1:** Dominant plant composition of different shrub plantations in micro-topography.

Micro-Topography	Species	Families	Form	Shrub Type
Windward slope	*Aster altaicus* Willd.	Asteraceae	HP	S, Z, H
	*Stipa capillata* L.	Poaceae	HP	S, Y, Z, H
	*Lactuca tatarica* L.	Asteraceae	HP	S
	*Corispermum hyssopifolium* L.	Amaranthaceae	AH	H
	*Setaria viridis* L.	Poaceae	AH	S
	*Cynanchum chinense* R. Br.	Apocynaceae	FU	S
	*Inula linariifolia* Turcz.	Asteraceae	HP	S
	*Agriophyllum pungens* Vahl.	Amaranthaceae	AH	Y, CK
Slope top	*Aster altaicus* Willd.	Asteraceae	HP	S, Y, Z, H
	*Stipa capillata* L.	Poaceae	HP	S, Y, Z, H
	*Lactuca tatarica* L.	Asteraceae	HP	S, H
	*Corispermum hyssopifolium* L.	Amaranthaceae	AH	Z, H
	*Setaria viridis* L.	Poaceae	AH	S, Y
	*Cynanchum chinense* R. Br.	Apocynaceae	FU	S
	*Echinops gmelinii* Turcz.	Asteraceae	HP	S
	*Grubovia dasyphylla* Fisch. & C. A. Mey.	Amaranthaceae	AH	S
	*Setaria faberi* R. A. W. Herrmann	Poaceae	AH	S
	*Agriophyllum pungens* Vahl.	Amaranthaceae	AH	Y, CK
Leeward slope	*Aster altaicus* Willd.	Asteraceae	HP	S, Y, H
	*Stipa capillata* L.	Poaceae	HP	S, Y, Z, H
	*Corispermum hyssopifolium* L.	Amaranthaceae	AH	Z, H
	*Setaria viridis* L.	Poaceae	AH	S
	*Cynanchum chinense* R. Br.	Apocynaceae	FU	S, Z
	*Inula linariifolia* Turcz.	Asteraceae	HP	H
	*Echinochloa crus-galli* var.	Poaceae	AH	S
	*Salsola collina* Pall.	Amaranthaceae	AH	S, Y, H
	*Teloxys aristata* L.	Amaranthaceae	AH	S
	*Suaeda glauca* Bunge.	Amaranthaceae	AH	S
	*Agriophyllum pungens* Vahl.	Amaranthaceae	AH	S, CK
Interdune lowland	*Aster altaicus* Willd.	Asteraceae	HP	S, Y, Z, H
	*Stipa capillata* L.	Poaceae	HP	S, Y, Z, H
	*Lactuca tatarica* L.	Asteraceae	HP	S
	*Corispermum hyssopifolium* L.	Amaranthaceae	AH	S, Z, H
	*Setaria viridis* L.	Poaceae	AH	Y, Z
	*Inula linariifolia* Turcz.	Asteraceae	HP	S
	*Thermopsis lanceolata* R. Br.	Fabaceae	HP	S
	*Grubovia dasyphylla* Fisch. & C. A. Mey.	Amaranthaceae	AH	S
	*Echinochloa crus-galli* var.	Poaceae	AH	Z, H
	*Salsola collina* Pall.	Amaranthaceae	AH	S, Y, H
	*Astragalus laxmannii* Jacq.	Fabaceae	HP	S
	*Sonchus brachyotus* DC.	Asteraceae	HP	S
	*Oxytropis myriophylla* Pall.	Fabaceae	HP	S
	*Ixeris chinensis* Thunb.	Asteraceae	HP	S
	*Astragalus melilotoides* Pall.	Fabaceae	HP	Z
	*Euphorbia humifusa* Willd. ex Schltdl.	Euphorbiaceae	AH	S
	*Onopordum acanthium* L.	Asteraceae	HP	S
	*Saussurea amara* L.	Asteraceae	HP	S
	*Cynanchum chinense* R. Br.	Apocynaceae	FU	S
	*Agriophyllum pungens* Vahl.	Amaranthaceae	AH	CK

Note: HP indicates herbaceous perennials; AH represents annual herbs; FU represents lianas. S represents *S. psammophila* monoculture; Y represents *C. fruticosum* monoculture; Z represents the monoculture of *A. fruticosa*; H represents S + Y + Z mixed plantation; CK represents bare sand.

**Table 2:** Two-way ANOVA and post hoc tests for soil nutrients.

Index	Variance Source	Sum of Squares (SS)	*df*	Mean Square (MS)	*F*	*p*	Post Hoc Test *
pH	MT	2.200	3	0.733	9.62	0.000 **	I > II, I > IV, III > IV
	Veg	116.625	4	29.156	382.38	0.000 **	S > Z, S > H, S > CK, Y > Z, Y > H, Y > CK, H > Z, Z > CK, H > CK
	MT × Veg	3.049	12	0.254	3.33	0.000 **	
SOM	MT	2.763	3	0.921	3.72	0.014 *	I > II, III > II
	Veg	44.459	4	11.115	44.92	0.000 **	S > Y, S > Z, S > H, S > CK, Z > Y, H > Y, CK > Y, Z > CK, H > CK
	MT × Veg	12.406	12	1.034	4.18	0.000 **	
TN	MT	512.086	3	170.695	22.30	0.000 **	II > I, IV > I, IV > II, IV > III
	Veg	2867.848	4	716.962	93.66	0.000 **	S > Y, S > Z, S > H, S > CK, Y > Z, Y > H, Y > CK, Z > CK, H > CK
	MT × Veg	251.647	12	20.971	2.74	0.003 **	
TP	MT	0.962	3	0.321	21.19	0.000 **	II > I, III > I, II > IV, III > IV
	Veg	6.262	4	1.565	103.47	0.000 **	Y > S, Z > S, H > S, S > CK, Y > H, Y > CK, Z > H, Z > CK, H > CK
	MT × Veg	0.173	12	0.014	0.96	0.496	
TK	MT	487.728	3	162.576	5.04	0.002 **	III > I, III > IV
	Veg	19,722.929	4	4930.732	152.83	0.000 **	Y > S, Z > S, S > H, S > CK, Y > Z, Y > H, Y > CK, Z > H, Z > CK, H > CK
	MT × Veg	1276.143	12	106.345	3.30	0.001 **	

Note: * indicates significant correlation (*p* < 0.05); ** indicates highly significant correlation (*p* < 0.01). MT denotes micro-topography, Veg denotes vegetational, and MT × Veg denotes interaction between micro-topography and vegetational. I indicates windward slope, II indicates slope top, III indicates leeward slope, and IV indicates interdune lowlands. S denotes *S. psammophila* monoculture, Y denotes *C. fruticosum* monoculture, Z denotes *A. fruticosa* monoculture, H represents S + Y + Z mixed plantation, and CK represents bare sandy land.

**Table 3:** Plot information table.

Plot Type	Longitude/°	Latitude/°	Elevation/m	Gradient/°	Windward Slope Direction	Height of Sand Dunes/m
*S. psammophila* (S)	109.247885	38.880868	1273.50	10	SW	7
*C. fruticosum* (Y)	109.202737	38.892425	1270.49	11	W	9
*A. fruticose* (Z)	109.235263	38.872782	1310.00	9	W	8
Mixed plantation (H)	109.235801	38.873037	1312.00	10	W	8
Bare sandy land (CK)	109.293522	38.808715	1276.68	11	SW	10

## Data Availability

The data presented in this study are available on request from the corresponding authors or first author. The data are not publicly available as the data are obtained from paid experiments.
